# MRI-based prediction of molecular subgrouping in medulloblastoma: images speak louder than words

**DOI:** 10.18632/oncotarget.27097

**Published:** 2019-08-06

**Authors:** Archya Dasgupta, Tejpal Gupta

**Affiliations:** ^1^ Department of Radiation Oncology, ACTREC, Tata Memorial Centre, Kharghar, Navi Mumbai, INDIA

**Keywords:** cradiogenomics, molecular subgrouping, medul-loblastoma, imaging

Medulloblastoma is the commonest malignant neoplasm of the central nervous system (CNS) in children [1] which arises in the posterior fossa and is classified histologically as World Health Organization (WHO) grade IV tumor. Novel biological insights have led to its consensus classification [2] into four molecular subgroups - wingless (WNT), sonic hedgehog (SHH), Group 3, and Group 4 medulloblastoma - each associated with distinct developmental origins, unique transcriptional profiles, diverse phenotypes, and variable clinical outcomes, which has now been incorporated in the 2016 update of the WHO classification of CNS tumors [3]. With rapid evolution of genomic technology, multiple platforms are now available for molecular subgrouping of medulloblastoma [4]. Accurate assignment of molecular subgroups mandates tumor tissue samples obtained during neuro-surgical resection, and usually involves complex molecular biology techniques such as gene expression profiling, microRNA analysis, or DNA methylation array [4]. Alternatively, expression of selected marker proteins on immunohistochemistry (IHC) - a much simpler technique with widespread availability in routine diagnostic pathology laboratories can be used for rapid molecular subgrouping of medulloblastoma [4].

Magnetic resonance imaging (MRI) is the preferred first-line imaging modality for patients with suspected brain tumors including medulloblastoma. Traditionally, pre-operative MRI has been used to arrive at a presumptive diagnosis based on lesion characteristics and extent of tumor spread. However, it is now being increasingly appreciated that imaging identifies and reflects underlying disease biology. Radio-genomics represents an exciting avenue of research that aims to correlate imaging-phenotypes with molecular/genetic markers [5]. In its simplest form, semantic imaging features (size, borders, intensity characteristics, contrast-enhancement, edema, necrosis, hemorrhage, calcification) can be assessed visually for correlation with molecular markers either singly or in combination. Introduction of computer-based algorithms has not only refined the assessment of semantic features, but also allows extraction of agnostic features (histograms, textures, wavelets, and fractal dimensions), thereby reducing time and virtually eliminating inter-observer variability associated with human interpretation.

Several features from pre-operative MRI have been used to find associations with specific molecular subgroups. In a large single-institution cohort of 111 patients [6] with known subgroup affiliation, a panel of 19 pre-specified semantic features on pre-operative MRI was used for correlation with molecular subgrouping. Multinomial logistic regression was performed on a training-set of randomly chosen 2/3rd of the patients to identify imaging features with highest discrimination of one subgroup from the other three subgroups to construct subgroup-specific binary nomograms, which was validated in the remaining 1/3rd of patients. The predictive accuracy of SHH-subgroup nomogram was the highest followed by Group 4-specific nomogram, while WNT-subgroup and Group 3-specific nomograms had suboptimal predictive accuracy. In a parallel study [7], 590 radiomic features (intensity-based histograms, tumor-edge sharpness, Gabor features, and local area integral invariant features) were extracted from T1- and T2-weighted MRI in a multi-institutional cohort of 109 children with medulloblastoma to identify four molecular subgroups using support vector machine algorithms. Acceptably high predictive accuracy of select radiomic features was reported for SHH and Group 4 medulloblastoma; however, radiomic signatures were much less robust for WNT-subgroup and Group 3 medulloblastoma. Based on results from both these studies, it can be inferred that SHH and Group 4 medulloblastoma can be reliably identified on routine pre-operative MRI sequences. However, the predictive accuracy for WNT and Group 3 medulloblastoma remains clearly suboptimal and unacceptably low for adoption in clinical practice.

**Figure 1:**
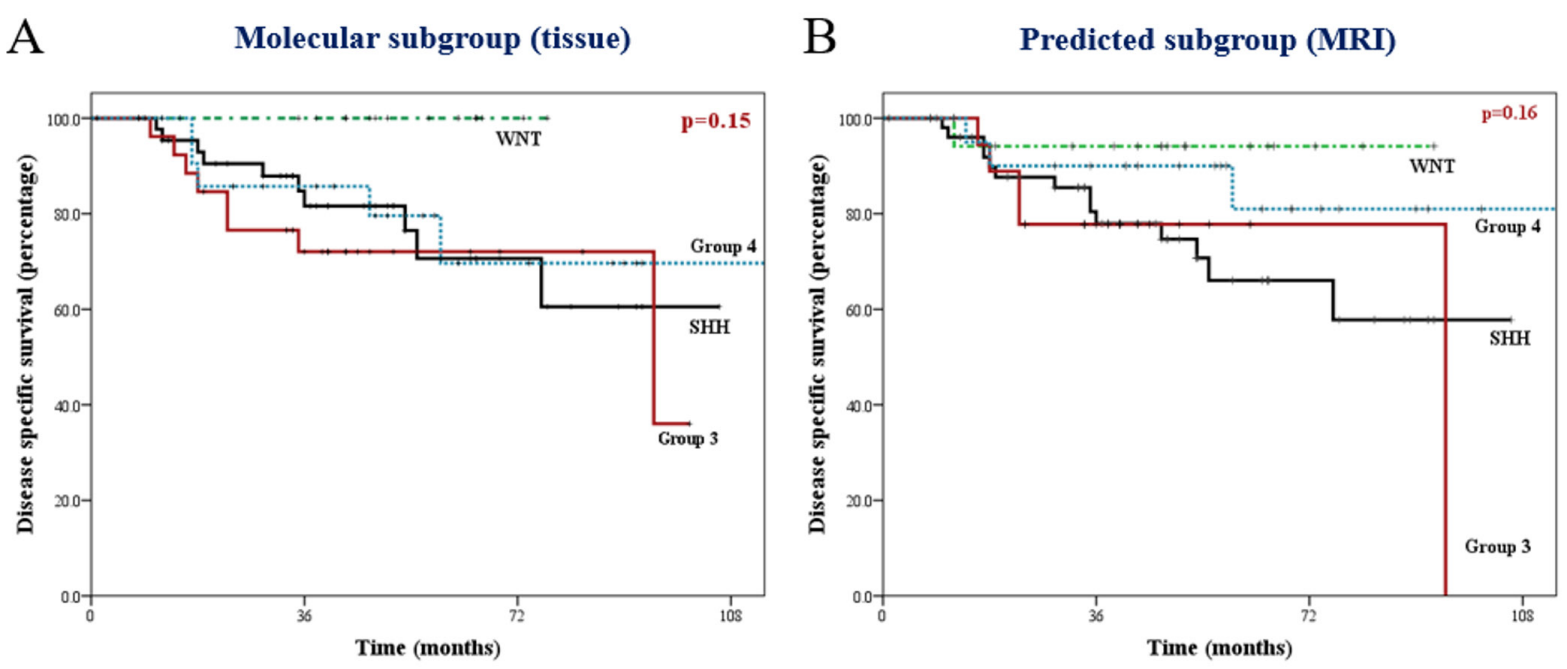
Kaplan-Meier curves of disease-specific survival (deaths due to disease progression) in patients with medulloblastoma Note the remarkable similarity between actual (tissue-based) subgrouping **A.**, versus imaging-predicted (MRI- based) subgrouping **B.**, across all four molecular subgroups.

Tissue-based information remains the gold-standard for histo-morphology and molecular subgrouping in medulloblastoma that cannot be replaced by any imaging-based classification. However, this tissue-based information is not available pre-operatively to the operating neuro-surgeon, but only after surgical resection of the tumor. The prognostic benefit of increased extent of resection becomes highly attenuated after taking molecular subgroup affiliation into account. In a retrospective multi-institutional cohort [8] involving 787 patients, there was no significant survival benefit of greater extent of resection (gross total/near total versus sub-total resection) for WNT, SHH, and Group 3 medulloblastoma. Only in Group 4 tumors, gross total/near total resection was associated with significant benefit in progression-free survival compared to sub-total resection, but not in overall survival. The authors concluded that although maximal safe resection should remain the standard of care, aggressive neuro-surgical removal of small residual portions of medulloblastoma should not be attempted, particularly when the anticipated morbidity is high. The availability of robust and reliable pre-operative imaging-based predictors of molecular subgrouping could therefore aid neuro-surgical decision-making. The addition of molecular genetic information in the 2016 update of the WHO classification [3] for an integrated diagnosis of medulloblastoma should ensure widespread adoption of a practical IHC-based classification in clinical practice. While this IHC-based classification identifies WNT and SHH subgroups with high accuracy, it is unable to reliably discriminate between Group 3 and 4 tumors which are classified collectively as non-WNT/non-SHH medulloblastoma with very differing prognosis. Imaging characteristics (contrast-enhancement of primary tumor and location, morphology, and pattern of metastatic disease) can further help to subclassify non-WNT/non-SHH tumors into Group 3 and Group 4 medulloblastoma with high degree of accuracy, particularly in resource-constrained settings. A comparison of disease-specific survival (Figure 1) between the actual molecular subgroups (tissue-based) and predicted molecular subgroups (MRI-based) from the author’s institutional dataset demonstrates remarkable similarity with retained discriminatory power. The contemporary standard of care in childhood medulloblastoma is post-operative craniospinal irradiation and tumor-bed boost followed by 6-9 cycles of platinum-based adjuvant systemic chemotherapy based on clinico-radiological risk-stratification (based on age, extent of resection, and leptomeningeal metastases). This has recently been supplanted by a more contemporary risk-classification schema (incorporating molecular biology) into low-risk, standard-risk, high-risk, and very high-risk categories [9] with potential of tailoring adjuvant therapy based on prognosis and expected long-term survival. In recent times, identification of significant intertumoral heterogeneity within individual medulloblastoma subgroups [10] has added another layer of complexity, posing further challenges to the radiologist that may be even more difficult to surmount. However, it is hoped that inclusion of newer and novel MRI sequences coupled with technological advancement and refinement of machine-learning approaches and artificial intelligence algorithms will overcome some of these challenges to further improve the predictive accuracy of such radio-genomic correlation.

## References

[1] Leece R (2017). Neuro Oncol.

[2] Taylor MD (2012). Acta Neuropathol.

[3] Louis DN (2016). Acta Neuropathol.

[4] Gupta T (2015). Curr Pediatr Rev.

[5] Kuo MD (2014). Radiology.

[6] Dasgupta A (2019). Neuro Oncol.

[7] Iv M (2019). Am J Neuroradiol.

[8] Thompson EM (2016). Lancet Oncol.

[9] Ramaswamy V (2016). Acta Neuropathol.

[10] Cavalli FMG (2017). Cancer Cell.

